# Perceived Impacts of a Public Health Training Center Field Placement Program among Trainees: Findings from a Small Group Externship Experience

**DOI:** 10.3389/fpubh.2014.00067

**Published:** 2014-07-04

**Authors:** Patrik Johansson, Brandon Grimm, Tarik Abdel-Monem, Stacey J. Hoffman, Mark DeKraai, Analisa McMillan

**Affiliations:** ^1^College of Public Health, University of Nebraska Medical Center, Omaha, NE, USA; ^2^University of Nebraska Public Policy Center, Lincoln, NE, USA

**Keywords:** public health training, public health training center, field placement, competency, HRSA

## Abstract

There is heightened interest in identifying the impact of the federally funded Public Health Training Center (PHTC) program. Although evaluation studies have been conducted of public health training in general, evaluations of PHTC programs are rare. Field placement components are congressionally mandated requirements of PHTCs. Field placements are typically intensive, supervised externships for students to gain public health experience with local health departments or non-profit organizations. We have found no published evaluations of PHTC field placement components. This may be because of their small size and unique nature. We designed and evaluated a 200-h field placement program at an established PHTC. The evaluation included pre/post surveys measuring public health core competencies, and post-experience interviews. We found significant increases in three competency domains among trainees: policy development and program planning, communication skills, and community dimensions of practice. These outcomes contribute to evidence based on the efficacy of PHTC field placement programs, and underscore their role in public health training.

## Background

Developing the public health workforce is a critical national priority. Workforce competencies and organizational quality have important implications for preparedness, community health surveillance and education, health system infrastructure, and overall health outcomes (1). Public health workforce development initiatives, however, remain underfunded while preventable population health concerns continue to grow (2). Evaluating workforce training initiatives play a critical role in making the case for further public health workforce development (3). However, the evidence base for evaluation of public health workforce training efforts is relatively small, though it continues to expand. Evaluations have been conducted on a range of public health training activities, including examinations of the impacts of evidence-based practices on individual skills (4, 5), trainings on disaster preparedness and response skills (6), leadership skills for upper-tier managers (7), community based participatory approaches to training (8), online and distance education-based training (9, 10), and satisfaction with formal Master’s in Public Health programs (11, 12). Public health researchers, practitioners, and program sponsors hope that continuing evaluation efforts will shed light on organizational capacity, service delivery, and, ultimately, consumer and public health outcomes (1, 13).

The federal government’s Public Health Training Center (PHTC) program plays an important role in workforce development. Established by Congress in 1998, the program provides graduate and specialized training in public health to improve preventive medicine, health promotion and disease prevention, and access to and quality of health services in medically underserved communities (14). Since its inception, 38 PHTCs have been created across the country at accredited schools of public health through a grants program administered by the U.S. Health Resources and Services Administration (HRSA). The program provides considerable autonomy for Training Centers to establish partnerships with state or local public health organizations to implement training activities. Evaluating the efficacy and impact of Training Center activities has thus become an important priority for this program. Of particular interest are documenting the impacts of field placement programs for students, which are a congressionally mandated requirement of Training Centers (15). Ultimately, thorough evaluation of the Training Centers and all their components will contribute to an evidentiary basis for understanding the impact of this program.

Experiential field placement programs are not uncommon in public health workforce training. Field placement programs are typically small in scale, as they involve direct funding for prolonged external placements of students. Master’s in Public Health programs commonly require field experiences as capstone practicums (16, 17), as do combined medicine and public health programs that merge community health and clinical experience training (18). Public health field placement programs build on a wider evidenced-based and theoretical body in experiential learning that values applied or clinical practice in real-world environments (19). Successful public health field placements provide trainees with opportunities for direct interactions with diverse sets of consumers and practitioners in applied settings (20), as well as exposure to managerial and fiscal considerations that influence most types of practice environments (21). An experiential supervisor helps mentor a trainee and direct a learning experience that is more socially and educationally supportive than a didactic environment (22, 23). Through experiential internships, trainees encounter real-world problems, are socialized into practice settings, and build self-confidence in their ability to practice in the field (24, 25).

Despite generally wide support for and continued administration of experiential field placement training programs within public health, there are relatively few evaluations of such activities. Studies by McIntosh, Block, Kapsak and Pearson (26), and Villanueva, Hovinga, and Cass (27) show high levels of trainee satisfaction with formal medical school and public health field internship programs. A study by O’Connell et al. (28) found that field internships can also impact trainee perceptions of health systems. However, beyond these works we found no other examples of evaluation studies that focus on individual impacts of PHTC-sponsored field placement programs. This may be because of the relatively small sizes of field placement programs, the multiple settings of placements, or the individualistic and qualitative nature of the educational experience; all factors, which complicate an evaluation design and limit generalization of results. Thus, beyond anecdotal data, little is known about the impact of field placement programs on trainee competencies and public health skills and knowledge in general. Nor is much known about the effect of field placement experiences on workforce development in the long-term, such as future practice intentions of trainees. However, because of the congressionally mandated funding of field placement programs as requirements of the PHTC, evaluations of these programs should be emphasized. This article outlines a description of a HRSA-sponsored Training Center program in Nebraska and results from a mixed-methods evaluation of its 200-h field placement program. The intention of this study is to contribute to the gap in knowledge about PHTC-sponsored field placement programs, and their perceived impacts and effectiveness.

## Program Description

The HRSA funded the Great Plains Public Health Training Center (Great Plains PHTC) at the University of Nebraska Medical Center College of Public Health, which was established in September 2011 (Award # UB6HP22821-01-03). The program targets urban and rural Nebraska and regional Tribal populations. The PHTC is dedicated to HRSA’s overall mission, *to strengthen the technical, scientific, managerial, and leadership competence of our existing and future workforce*. The goals of the Great Plains PHTC are to: (1) establish an effective, durable infrastructure to sustain the Great Plains PHTC, including its advisory council, domain-specific working groups, administrative leadership and staffing, working logic models, and timelines; (2) conduct comprehensive assessments of workforce development and training needs; (3) increase and strengthen the technical, scientific, managerial, and leadership capacity in underserved Nebraska and Tribal communities through workforce development and training, in alignment with the Council on Linkages, Core Competencies for Public Health Professionals; (4) promote and support selected collaborative projects between faculty and students and targeted communities in underserved Nebraska and Tribal areas; (5) provide and promote field placements in identified priority populations; and (6) assure rigorous PHTC evaluation and quality improvement. The Great Plains PHTC addresses these goals through governmental, academic, Tribal, and regional collaboration; systematic assessment of needs; competency-based adult learning and workforce development, leadership and management training; field placements and collaborative projects; and evaluation and quality improvement.

In 2011–2012, the Great Plains PHTC initiated a 200 h field placement program for the first cohort of students. The field placement experience lasted over a 3-month-time frame in the summer of 2012. Designed to link trainees with local or tribal health departments in Nebraska in need of assistance with public health-related projects, the field placements could offer practical and meaningful guidance through a designated site supervisor and a Training Center mentor. The goal of the experiences was to give students the hands-on chance to experience social determinants of health, broaden their cultural awareness, and tackle everyday issues that affect the public’s health. Field placement training experience topics ranged from tobacco/alcohol/drug prevention to farm safety to community needs assessment projects. Field placement experiences were intentionally designed to expose trainees to clients and populations the training site typically served, and provide trainees with insight into program development and implementation. Three of the placement sites specifically geared their experiences toward working with racial and ethnic minority populations. As part of the program structure, trainees were asked to reflect on and discuss their experiences working with communities and in programs with each other and their supervisors on a regular basis through conference calls and in-person meetings.

There were a total of 10 field placement trainees. Two of the trainees were also enrolled as Master’s level students in public health and epidemiology. Seven of the students were pursuing undergraduate degrees (two in biological sciences, two in social sciences, and one each in nursing, pre-pharmacy, and one in sports exercise). Three of these undergraduate students were considering continuing their studies to obtain a Master’s in Public Health, one was considering an advanced nursing degree, and the rest were undecided. Finally, one student had received an undergraduate degree in nursing and was contemplating a Master’s in Public Health. Nine of the 10 field placements were conducted in rural communities across the state, and one in the Omaha metropolitan area. Nine of the field placements were located at local governmental public health departments, and the remaining internship was held at an urban clinic, which served Native Americans. Seven of the 10 field placement sites identified their locations as health professions shortage areas. Additionally, three locations indicated that they served medically underserved communities.

## Methods

Due to the small size of the field placement class, we used both surveys and interviews in our evaluation approach. Interview content would augment and explain survey data so a more well-rounded and reliable sense of trainees’ perceptions could be discerned. A pre–post survey design was implemented. Surveys were administered prior to the trainees beginning their field placement, and following the completion of their 200-h internship. Interviews were then conducted at the completion of their internship.

### Survey procedures

All eight Council on Linkages’ public health core competency skill domains were featured in the pre and post-field placement survey: analytic/assessment, policy development and program planning, communication, cultural competency, community dimensions of practice, public health science, financial planning and management, and leadership and systems thinking skills. The core competency domains were selected as criteria for measurement because of the competency-based emphasis of the Training Center program, and their acceptance within the public health practice field generally. In addition to these core competency domains, we also developed and included competency items for two additional domains: HIV/AIDS-related programing skills, and tribal health-related skills. The post-training survey also featured questions asking trainees to assess satisfaction of their field placement experiences, and questions about their intentions to practice in three settings: primary care, medically underserved, and rural.

### Interview procedures

Seven field placement trainees participated in telephone interviews with the researchers within a 2-week period of finishing their field internship, and following their completion of post-field placement surveys. The field placement trainees were provided with IRB-approved informed consent before participating in the interviews. Trainees were asked seven open-ended questions about their field placement experience (see Appendix), in addition to several follow-up probing questions if appropriate. The telephone interviews lasted between 10 and 30 min, and all interviews were audio recorded and transcribed with the permission of the trainees.

Interview content was analyzed using a grounded theory, constant comparison approach (29, 30). Interview transcriptions were reviewed independently by two of the authors. ATLAS.ti software was used to assist with analysis and coding. Broad themes emerged from the interview questions and responses, and thematic codes were implemented to identify relevant quotations following discussion by the authors (31). A standard coding definition format was used in order to be consistent with identifying themes (32). Following independent review, the authors discussed codes to rectify discrepancies and reach agreement on themes.

## Survey Results

### Demographics, personal background, and intentions for practice

All 10 participants completed the training center’s field placement experience during the summer of 2012. Basic demographic information from participants is presented in Table 1. A majority (80%) of the participants were women; fell into the typical age group for college juniors through public health Master’s-level students (70% were age 20–29 years); and were white (70%) and non-Hispanic (80%), which generally reflects the ethnic make-up of the state of Nebraska. Participants represented a range of educational levels, including high school graduates, and those with Associates, Bachelors, and Master’s Degrees (see Table 1).

**Table 1:** **Demographics and participant background as a percentage of the sample**.

Characteristic	*n* = 10
**GENDER**	
Male	20.0
Female	80.0
**AGE GROUP**	
Under 20 years	10.0
20–29 years	70.0
30–39 years	10.0
40–49 years	–
50–59 years	10.0
60–69 years	–
70 years or older	–
**RACE**	
American Indian or Alaska native	10.0
Asian	–
Black or African-American	–
Native Hawaiian or other pacific islander	–
White	70.0
Unknown	10.0
More than one race	10.0
**ETHNICITY**	
Hispanic or Latino	20.0
Non-Hispanic or Latino	80.0
**HIGHEST EDUCATIONAL LEVEL COMPLETED OR DEGREE**	
Grades 9–12	30.0
Post-high school/pre-college	10.0
Associates	10.0
Bachelors	20.0
12-month post-baccalaureate	10.0
Master’s	10.0
Doctorate	–
Post-doctorate	–
**PROFESSION**	
Medicine	20.0
Public health	80.0
**DISADVANTAGED BACKGROUND**	
Yes	30.0
No	70.0
**RESIDENTIAL BACKGROUND**	
Rural	50.0
Suburban	30.0
Urban	20.0

Field placement students were asked to indicate the profession they considered best described their work. Eighty percent indicated that they considered themselves to be in the Public Health field, while 20% considered themselves to be in Medicine. Those who reported their profession as public health were equally likely as those who reported their profession as medicine to indicate they would practice in a primary care setting [χ^2^(2) = 1.88, *p* = 0.392].

When asked about their personal background, 30% of participants reported being from a disadvantaged background, while 70% reported they were not from a disadvantaged background^1^. Those who reported they had a disadvantaged background were more likely to intend to practice in a medically underserved community [χ^2^(2) = 6.43, *p* = 0.040].

Fifty percent of participants were from a rural residential background, 30% from a suburban background, and 20% from an urban background. Those who reported growing up in an urban setting reported they would not practice in a rural setting, while those from a suburban or rural background indicated they were unsure [χ^2^(2) = 10.00, *p* = 0.007].

### Ratings of training experience

At the end of their field placement, participants rated the experience on a few dimensions. On a scale of 1–5, with “1” being “Strongly Disagree” and “5” being “Strongly Agree,” participants tended to agree that their knowledge increased (*M* = 4.00, SD = 1.25), and also agreed that they would recommend the training to others (*M* = 4.44, SD = 0.53). They also tended to rate the experience as being either fairly easy (44.4%), or challenging but not too difficult (44.4%). One participant thought that the experience was too easy (11.1%), while none found it too difficult.

Participants were also asked to indicate their preference for a variety of learning formats and the importance to them of several characteristics of public health training (see Table 2). For preference of learning formats, participants used a 1–5 scale, with “1” being “Not Preferred At All” and “5” being “Very Preferred.” The most preferred learning format was an in-person training session lasting 1 day or less (*M* = 4.11, SD = 0.78). For importance of training characteristics, participants used a scale of 1–5 on which “1” was “Low Importance” and “5” was “High Importance.” The most important characteristic was the opportunity to interact face-to-face with an instructor (*M* = 4.70, SD = 0.48).

**Table 2:** **Preferences for learning formats and importance ratings of training characteristics**.

Item	*M*	SD
**PREFERENCE FOR LEARNING FORMATS^a^**		
In-person training session (1 day or less)	4.11	0.78
In-person training session (multi-day workshop)	3.56	0.88
Live training via distance learning	3.56	1.01
College or university course work with or without credit	3.56	1.01
Online training at your own pace	3.22	1.39
Non-online training at your own pace	2.89	1.36
**IMPORTANCE OF TRAINING CHARACTERISTICS^b^**		
Opportunity to interact face-to-face with an instructor	4.70	0.48
Flexible timing (learning in the evenings or weekends)	4.50	0.53
Availability of financial aid	4.50	0.85
Support from employer/superiors to attend training	4.20	0.79
Availability of continuing education credits	4.20	0.92
Opportunity to complete training at your own pace	4.20	0.92
Opportunity to interact face-to-face with other participants	4.20	1.03
Being part of a group with students at different levels of experience	4.10	0.99
Being part of a group that is at the same learning level	4.10	1.20

^a^Preference for learning formats was rated on a scale from 1 to 5, with “1” being “Not Preferred At All” and “5” being “Very Preferred.”

^b^Importance of training characteristics was rated on a scale from 1 to 5, with “1” being “Low Importance” and “5” being “High Importance.”

### Comparison of public health core competencies pre-training vs. post-training

Ideally, we would have liked to use MANOVA to compare differences across time on the public health core competencies. However, the small sample size did not provide a large enough number of degrees of freedom to conduct this analysis for most of the core competency sets of items. Eight participants completed all 10 of the core competency questions before and after the training experience. Six of the 10 core competencies included eight or more items, and two included six items. Due to this limitation, we calculated each participant’s average pre-training and post-training scores on each of the core competencies. We then conducted a series of 10 simple ANOVAs to compare the pre-training and post-training scores for each core competency. Doing so, we found significant increases in knowledge on three domains, even given the small sample size: Program Development and Program Planning Skills [*M*_Pre_ = 2.69, SD_Pre_ = 0.99; *M*_Post_ = 3.49, SD_Post_ = 0.60; *F*(1, 9) = 7.46, *p* = 0.023]; Communication Skills [*M*_Pre_ = 2.92, SD_Pre_ = 0.98; *M*_Post_ = 3.88, SD_Post_ = 0.74; *F*(1, 9) = 5.51, *p* = 0.044]; Community Dimensions of Practice [*M*_Pre_ = 3.01, SD_Pre_ = 1.33; *M*_Post_ = 3.89, SD_Post_ = 0.51; *F*(1, 9) = 5.77, *p* = 0.040]. Public health core competency scores increased from pre-training to post-training across all domains except one (HIV/AIDS-related programing skills), but increases were significant only for the aforementioned three domains. See Table 3 for the means, standard deviations, and significance tests of all competency domains.

**Table 3:** **Comparison of public health core competency skills before and after the training experience**.

Core competency domain	Pre-training		Post-training		*F*	*p*
	*M*	SD	*M*	SD	
Analytic/assessment	3.01	1.09	3.65	0.67	3.17	0.109
Policy development and program planning	2.69	0.99	3.49	0.60	7.46	0.023^a^
Communication	2.92	0.98	3.88	0.74	5.51	0.044^a^
Cultural competency	3.37	1.36	4.24	0.80	3.30	0.107
Community dimensions of practice	3.01	1.33	3.89	0.51	5.77	0.040^a^
Public health sciences	3.11	1.16	3.57	0.75	1.89	0.207
Financial planning and management	2.68	1.24	3.25	0.62	3.14	0.110
Leadership and systems thinking	3.55	1.31	3.59	0.75	0.02	0.902
HIV/AIDS-related programing	3.33	0.92	3.20	1.46	0.08	0.778
Tribal health	2.48	1.22	2.75	1.11	0.37	0.558

*^a^The post-training mean is significantly larger than the pre-training mean, indicating increased knowledge on this domain*.

## Interview Results

We summarized interview content into several areas. Participants believed there were two general aspects of their placement experiences, which were valuable: *broad exposure to public health practice*, and *meaningful community engagement*. We also summarized perceptions of trainees’ *skills improvement and self-efficacy* as a result of the experience. Trainees also offered generally similar recommendations for *program improvements*, both for administration of the field placement program, as well as general thoughts on host sites.

### Broad exposure to public health practice

Several interviewees noted that there was significant value in field placement experience, which provided them with extensive exposure to a variety of public health practice activities. Because most of the interviewees had not had previous exposure to public health outside of the classroom, the field placement served as an opportunity to understand the array and scope of skills and activities in public health practice. A number of individuals believed that this exposure helped them to better understand the field, and their particular interests and abilities. The field placements served to fulfill a basic role of exposing students to problems and activities not available in the classroom, how they are dealt with on an inter-organizational and community level, and socialized students to the culture and values of public health practice:

I’d say mostly, the biggest thing I learned was how big public health was. I got to see something with workplace wellness, stuff I helped with the data, and then I got to help with a couple of community events that they sponsored and just saw how much they really do. I attended a bunch of meetings, like their board of health meeting. I just never knew that public health was that big or that it was that prominent in communities, even in my community where I have lived forever. I guess that was one of the biggest things I learned.

Interviewees noted that exposure to broad sets of activities helped them feel better prepared to continue graduate studies in the field, and was thus a positive motivational influence to learn more. Several trainees indicated that the placement experience helped shed light on interdisciplinary connections between public health and other forms of healthcare practice:

I am applying to accelerated nursing programs for now and I will see where that takes me. What I realized this summer is that the nursing program really coincides with public health a lot of times, especially with our clinic, we had two RNs and a nurse practitioner, so that was really great to see how public health and nursing were tied in together.

I think having this background in public health is extremely helpful. You don’t see a whole lot of pharmacists familiar at all with public health as of yet but I know that at [the college of public health] they are thinking about making a joint program to have your masters of public health and be a pharmacist so it is something that is up and coming, I’m actually going there at the end of this month, I plan to ask the people there what is going on with that program.

Exposure to interdisciplinary connections in the field thus helped students understand how different but related academic fields could be applied in cooperative efforts. It also served as an opportunity for trainees to sharpen their awareness of and interests in future career paths.

### Meaningful community activities

All of the interviewees indicated that they participated in numerous community interaction activities, particularly health promotion and education events. Interviewees noted that they found great value in assisting with projects that had “real impact” on the communities in which they were based, as opposed to traditional classroom activities. They also indicated that they were appreciative of activities, which involved use of different skills in community-focused activities. For instance, several of the trainees were specifically assigned to community health assessment projects using the Mobilizing for Action through Planning and Partnerships (MAPP) framework, an assessment and evaluation process that requires data gathering and analysis and public engagement (33). Interviewees noted that they enjoyed using multiple methods to gage community health status, from direct interaction and engagement, to review of data from existing sources:

I attended focus groups. Part of the community assessment was to ask different parts of the community general questions about health, and I added it to my community health assessment report…. I helped them look in databases like vital statistics or the behavioral risk factor survey and I collected data for all their indicators so they could start their community health status assessment, which I think they are pretty well established with now. I have taken stats, so I remember stats and confidence intervals and determining if data is significant or not, and interpreting that data. I think that was one of the biggest things I gained as a new skill.

Several of the field placements involved direct participation in community health interventions. These interventions ranged from supporting community health promotion and behavior initiatives, to providing preventative education in clinical settings. This included participating in evidenced-based interventions that allowed them to have direct contact with communities of interest:

My main project for the summer was working with the Reward and Reminder program, which is doing tobacco compliance checks in area grocery stores and gas stations. We took in minors, and then clerks were rewarded with a subway gift card if they terminated a sale with a minor after ID’ing them and knowing that they either do not have their ID on them or knowing that they were underage…. It was just positive in getting experience in a field that I know most of us had not been exposed to.

Two of the interviewees had nursing education backgrounds. They assisted with clinical activities, and were thus able to interface with consumers in primary care environments. These interviewees indicated that working with actual clients had a motivational effect on them because they could work directly with people in need. For instance, one of the trainees spent significant parts of her placement experience working with children and families in need of services:

A lot of times we had children come in that were under nourished. We documented that and we tried to educate the parents. This happened a lot, there is a large Hispanic population that came in to work around there. We tried to educate them about proper nutrition for their children so their children weren’t going hungry, and that was a big part of how we helped people. Just the patient education. I guess that was probably the main aspect.

### Skills improvements and self-efficacy

The majority of interns noted that the field placement provided them with specific opportunities to learn and practice new skills, which improved perceptions of their abilities and their self-confidence. This seemed particularly relevant with interns who had nursing backgrounds, and had participated in internships in clinical settings. This helped trainees make connections between clinical services in the primary care setting, and how it is tied to wider public health missions:

I did some in home visits with the public health nurses for expecting females. I did in home visits for individuals with high blood pressure, high cholesterol. The nurses would give them a good diet plan and exercise regimen…. I did learn a lot of hands-on stuff about missing immunizations and proper techniques for drawing blood, especially for multiple procedures and that was really fun. I also learned about what public health really is and what the government and the state does to aid in public health and how much public health really is available.

Interviewees acknowledged skills-gains in other areas, including in basic public health skills like statistics and epidemiology, policy analysis, general research, and communication. One interviewee remarked how her experience working with community members increased her sense of confidence with social interaction generally:

I’m not really a very open person you know personally. Like talking or you know, some people are really popular because they talk with everybody and they have fun and go out and all that stuff. But I’m not really that kind of person and it was really surprising even for me that I was able to do this meeting, and I really felt engaged with doing it….I said wow, I can talk to people…. They really were so happy because the meeting I organized with the community was so successful, and people participated.

### Program improvements

Trainees did have several insights into how the field placement program could be improved. Most suggestions were administrative in nature, with trainees recommending more opportunities for structured interactions and expectations with program staff. Specific recommendations included having a greater number of in-person meetings between program staff, placement site hosts, and trainees during the placement experience; having detailed lists of well-defined objectives for trainees in place; reducing or eliminating office administrative tasks for trainees; providing financial support specifically to assist with transportation; providing more rigorous assignments or evaluations for trainees during and after their experiences.

It should be noted that three of the placement sites intentionally assigned students to assist with community outreach activities directed toward ethnic and racial minorities. Thus the need to expand connections to minorities is a recognized priority among health departments and organizations in Nebraska. One field placement student of Latina background reported that as a result of her traineeship, she was pursuing plans to volunteer further with her assigned placement site, a rural health department in central Nebraska. The field placement program thus did lead to at least one potential post-program connection that will help fill needs in these areas.

## Discussion and Limitations

Public health student field placements represent a core activity of PHTC. In our evaluation of the newly established Great Plains PHTC’s field placement program, we found significant self-reported increases in three competency domains: policy development and program planning, communication skills, and community dimensions of practice. These increases reflect field placement experiences that emphasize meaningful exposure to and interaction with communities and clients, and opportunities to better understand program structures and implementation in real-world settings – all elements, which were emphasized in the field placement program experience. In considering the overall impact of field placement programs on trainees, these results thus indicate that such activities can have a meaningful immediate impact on perceived skills of trainees. Such findings support the assertion that public health field placements advance the learning of trainees by providing them with applied and practice-based educationally and socially supportive experiences.

Among our sample of trainees, those who came from a disadvantaged background reported that they were more likely to intend to practice in a medically underserved community. This finding supports existing literature that demonstrates that individuals from racial and ethnic minority and disadvantaged backgrounds are more likely to practice in underserved communities (34, 35). This finding is important as organizations such as the Institute of Medicine, the Association of Schools of Public Health, the Association of American Medical Colleges, and other leading entities support diverse student bodies with representation from disadvantaged backgrounds as a means to address health profession shortages in underserved communities (36, 37). Because a number of the field placement experiences convened under the Great Plains PHTC involved working with underserved or minority populations, it is possible that the field experiences contributed to trainees’ intentions to work with underserved communities in the future.

There are some limitations with this study that should be acknowledged. Because of a small sample size, the types of quantitative analyses that could be used were limited, and their results should be interpreted with caution. Analyses requiring a large number of degrees of freedom could not be conducted. Thus, simple comparison of means was used to examine differences in core competencies from the beginning to the end of training. Furthermore, the sample size also limited the ability to detect smaller scale differences across time in the core competencies. While we were able to detect differences that were large, there may be medium or small differences that we were unable to detect due to lack of power. Larger sample sizes would increase the likelihood that smaller pre/post changes could be detected, and that results could be more generalizable. However, it must be noted that field placement programs generally are small in size due to the amount of funding required to support prolonged externships in the field. We believe one of the reasons why there exists an absence of published evaluations of field placement programs is due to their small size, which hinders meaningful statistical analyses. Despite their small size, we believe that evaluating these field placement experiences is important not only because of their overall prevalence in public health training, but because they represent specific requirements of the HRSA-funded PHTC program (15). Augmenting our evaluation with qualitative analysis helps provide insight where quantitative analysis is limited. Recognizing that results are limited by the small *N*, a mixed-methods approach, such as we have employed, represents one viable way to evaluate this small field placement program and provide additional context to any findings.

## Conclusions and Directions for Future Research

Our study contributes to the evidence based on the efficacy of PHTC field placement programs, and underscores the important impact that an intensive student field placement program can have in enhancing public health practice competencies among trainees and addressing future public health profession needs where shortages exist. However, further research is needed about what particular elements of field placement experiences or characteristics impact competencies and practice intentions among trainees. Additionally, future studies should be conducted to identify the influence of the program beyond impacts on trainees, and focus on organizational impacts, service delivery and programing, and discernible community impacts. As resources are tightened at local and state public health departments around the country, it is important that training activities like the PHTC field placement program do not place added burdens on the placement sites. Future research should be conducted to explore whether the benefits outweigh the costs (time to train, physical space, supervision, etc.), in addition to the long-term impact of student placements in underserved areas in addressing public health professions shortages in such communities. As grant funding continues to decrease, it is important for public health practice researchers to continue to provide evidence of effectiveness and return on investment of programs such as this.

In considering future directions for public health field placement programs, the Institute of Medicine’s report on primary care and public health recommends that HRSA create specific Title VII and VIII criteria or preferences related to curriculum development that favor the integration of public health and primary care (37, 38). Students who participated in the PHTC program described themselves as either working in public health or medicine, and those who reported their profession as public health were equally likely as those who reported their profession as medicine to indicate they would practice in a primary care setting. Based on the findings from our study, as HRSA creates training programs to prepare coming generations of public health and other health professionals, field placements should be part of the consideration of such public health/primary care integration efforts.

## Conflict of Interest Statement

The authors declare that the research was conducted in the absence of any commercial or financial relationships that could be construed as a potential conflict of interest.

## Footnotes

^1^This study used the Health Resources and Services Administration (HRSA) definition of disadvantaged background: an individual who (1) educational comes from an environment that has inhibited the individual from obtaining knowledge, skills, and abilities required to enroll in and graduate from a health professions school; or (2) economically comes from a family with an annual income level below certain levels based on family size.

## Appendix

### Interview Questions

What is your professional and/or academic background in health or public health?

How did you first hear of this field placement opportunity?

Where was your field placement, and what did you do?

What was your overall assessment of your field placement experience?

What new skills or bodies of knowledge did you learn from this field placement experience?

How do you plan to apply your field placement experience to future plans?

Do you have any suggestions for improving the field placement experience?
